# Reduction of White Spot Defects in CMOS Image Sensors Fabricated Using Epitaxial Silicon Wafer with Proximity Gettering Sinks by CH_2_P Molecular Ion Implantation

**DOI:** 10.3390/s22218258

**Published:** 2022-10-28

**Authors:** Takeshi Kadono, Ryo Hirose, Ayumi Onaka-Masada, Koji Kobayashi, Akihiro Suzuki, Ryosuke Okuyama, Yoshihiro Koga, Atsuhiko Fukuyama, Kazunari Kurita

**Affiliations:** 1 SUMCO Corporation, 1-52 Kubara, Yamashiro-cho, Imari-shi, Saga 849-4256, Japan; 2 Faculty of Engineering, University of Miyazaki, 1-1 Gakuen-kibanadai-Nishi, Miyazaki 889-2192, Japan

**Keywords:** CMOS image sensor, molecular ion, white spot defects, gettering

## Abstract

Using a new implantation technique with multielement molecular ions consisting of carbon, hydrogen, and phosphorus, namely, CH_2_P molecular ions, we developed an epitaxial silicon wafer with proximity gettering sinks under the epitaxial silicon layer to improve the gettering capability for metallic impurities. A complementary metal-oxide-semiconductor (CMOS) image sensor fabricated with this novel epitaxial silicon wafer has a markedly reduced number of white spot defects, as determined by dark current spectroscopy (DCS). In addition, the amount of nickel impurities gettered in the CH_2_P-molecular-ion-implanted region of this CMOS image sensor is higher than that gettered in the C_3_H_5_-molecular-ion-implanted region; and this implanted region is formed by high-density black pointed defects and deactivated phosphorus after epitaxial growth. From the obtained results, the CH_2_P-molecular-ion-implanted region has two types of complexes acting as gettering sinks. One includes carbon-related complexes such as aggregated C–I, and the other includes phosphorus-related complexes such as P_4_–V. These complexes have a high binding energy to metallic impurities. Therefore, CH_2_P-molecular-ion-implanted epitaxial silicon wafers have a high gettering capability for metallic impurities and contribute to improving the device performance of CMOS image sensors. (This manuscript is an extension from a paper presented at the 6th IEEE Electron Devices Technology & Manufacturing Conference (EDTM 2022)).

## 1. Introduction

Complementary metal-oxide-semiconductor (CMOS) image sensors have been widely used not only for imaging using digital still cameras, smartphones, and others, but also for sensing in, for example, automobiles and security systems with the progress of the internet-of-things (IOT) society. Among them, three-dimensionally stacked back-side-illuminated CMOS image sensors (3D-CISs) products have been actively developed to achieve the desired characteristics, such as high resolution, high sensitivity, and high-speed imaging data processing [1]. However, 3D-CISs have serious technological issues that degrade device characteristics, such as dark currents and white spot defects associated with the device fabrication process [2].

The first important technological issue is metallic impurity contamination in the device active region during 3D-CIS fabrication processes, such as nickel silicide formation, electrode material deposition, and interconnection formation. Metallic impurity contamination tends to be enhanced because pixel dies are stacked on the signal processing circuit die using 3D-CIS integration technologies, such as Cu-through-silicon vias (Cu-TSVs) and Cu–Cu hybrid bonding [3,4,5]. Metallic impurities introduced into the depletion layer of a photodiode localize and form deep-energy-level defects in the silicon band gap. As a result, the number of white spot defects increases owing to the dark currents generated through the deep-energy-level defects existing in the depletion layer [6,7].

The second important technological issue is the out-diffusion of oxygen impurities from a Czochralski (CZ) silicon substrate to the depletion layer of a photodiode in pixels [8]. The oxygen impurities form deep-energy-level defects that act as generation–recombination (G–R) centers in the depletion layer. Thus, it is important that the region forming the photodiode has a low oxygen concentration.

The third important technological issue is the interface state densities (D_it_) at the SiO_2_/Si interface formed in the deep trench isolation (DTI) region or at the bonding interface of 3D-CIS [9]. The interface area in the DTI region increases with the number of pixels, and a bonding interface is also generated between the pixel die and signal processing circuit die of the 3D-CIS structure. The origin of D_it_ is the Si dangling bonds existing at the SiO_2_/Si interface (P_b_ centers [10,11]), which act as G–R centers owing to irregular trap and release carriers. Thus, dark currents are generated through the SiO_2_/Si interface [2].

Generally, low-temperature hydrogen forming gas annealing (FGA) at the back end of the line (BEOL) process is one of the methods of passivating P_b_ centers and reducing the D_it_ [10,12,13]. However, in the case of the 3D-CIS fabrication process, multi-dielectric films are often used in metallic wire deposition in the pixel region. Hydrogen cannot easily diffuse to the SiO_2_/Si interface owing to the multi-dielectric films acting as a barrier during FGA [2,12,13,14]. Therefore, a functional silicon wafer that can overcome these important technological issues is required.

**Figure 1:**
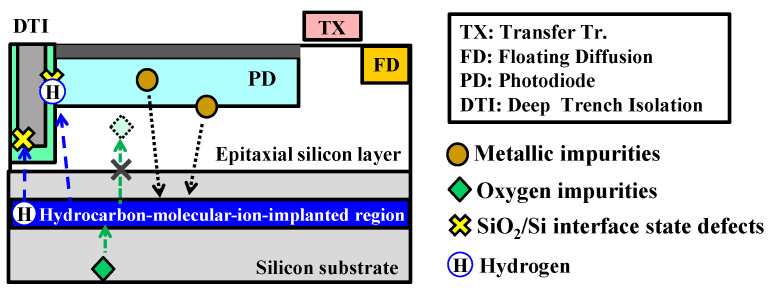
Three characteristics of hydrocarbon-molecular-ion-implanted epitaxial silicon wafer for CMOS image sensor fabrication process.

To realize this, we have developed an epitaxial silicon wafer with functional proximity gettering sinks introduced under the epitaxial silicon layer using the hydrocarbon (C_3_H_5_) molecular ion implantation technique [15,16,17,18,19,20]. In our previous research, we found that a C_3_H_5_-molecular-ion-implanted region has three characteristics that can resolve these technological issues, as shown in Figure 1. First, this ion-implanted region has the gettering capability for metallic impurities. Second, this ion-implanted region also acts as a diffusion barrier to the device active region from the silicon substrate because of the trapping of oxygen impurities during the CMOS image sensor fabrication process. Third, there is a passivation effect on D_it_ utilizing the hydrogen trapped in the C_3_H_5_-ion-implanted region after epitaxial growth and to diffuse during the CMOS image sensor fabrication process [21,22,23,24].

Kurita and coworkers demonstrated that the three characteristics of C_3_H_5_-molecular-ion-implanted epitaxial silicon improved electrical performance, such as the reduction in the number of white spot defects and dark currents in CMOS image sensors as determined by dark current spectroscopy (DCS) [25,26]. DCS is a powerful metallic impurity contamination analysis method, which enables us to count generated dark currents in pixels in charge-coupled devices (CCDs) and CMOS image sensors [7,27,28,29].

Furthermore, we developed a new implantation technique with multielement molecular ions consisting of carbon, hydrogen, and phosphorus, namely, CH_2_P molecular ions, with the aim of improving the gettering capability for metallic impurities among the three characteristics to further reduce the number of white spot defects in CMOS image sensors. As a gettering technique using phosphorus, high-density misfit dislocations and the P–V complex called E-centers are formed in the high-phosphorus-concentration region using thermally diffused phosphorus and phosphorus monomer ion implantation from the back surface of the silicon wafer; and they act as gettering sinks for metallic impurities [30,31,32,33]. In this study, we characterized the gettering capability of the CH_2_P-molecular-ion-implanted epitaxial silicon wafer by comparing the number of white spot defects obtained by the DCS of the CMOS image sensor with a C_3_H_5_-molecular-ion-implanted epitaxial silicon wafer.

## 2. Materials and Methods

**Figure 2:**
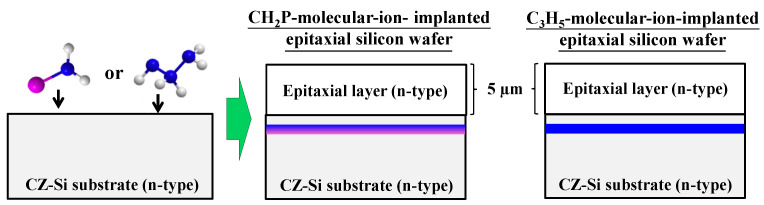
Cross-sectional structures of CH_2_P- andC_3_H_5_-molecular-ion-implanted epitaxial silicon wafers.

Figure 2 shows the cross-sectional structures of the epitaxial silicon wafers used in this study. The samples were n-type CZ-silicon wafers doped with carbon. The phosphorus concentration was 6.7 × 10^14^/cm^3^, the carbon concentration was 4.7 × 10^16^/cm^3^, and the initial oxygen concentration was 14.5 × 10^17^/cm^3^ (old ASTM). The silicon wafer surface was implanted with CH_2_P molecular ions using CLARIS (Nissin Ion Equipment). The implantation conditions were an energy of 80 keV/molecule and a dose of 2.0 × 10^14^ ions/cm^2^ (carbon, hydrogen, and phosphorus doses were 2.0 × 10^14^ atoms/cm^2^, 4.0 × 10^14^ atoms/cm^2^, and 2.0 × 10^14^ atoms/cm^2^, respectively). The tilt and twist angles were both set to 0°. For the comparison of the number of white spot defects, C_3_H_5_-molecular-ion-implanted silicon wafers were prepared using the same energy (80 keV/molecule) and carbon dose (2.0 × 10^14^ atoms/cm^2^) as those for CH_2_P molecular ion implantation. After molecular ion implantation, the thickness of the epitaxial silicon layers deposited on the silicon surface by chemical vapor deposition was 5.0 µm. Subsequently, we fabricated a CMOS image sensor with four transistors in a pixel with a pinned photo diode using the CMOS device fabrication process.

We measured the number of white spot defects in each image sensor implanted with CH_2_P and C_3_H_5_ molecular ions by DCS. After that, these sensors and epitaxial silicon wafers were evaluated by the methods described below to confirm the difference in the number of white spot defects between the molecular-ion-implanted samples. The size and density of bulk microdefects (BMDs) were measured using a BMD analyzer (MO-441^®^, Optima Incorporated, Kanagawa, Japan). The concentration profiles of carbon, oxygen, phosphorus, and nickel in the depth direction were analyzed by secondary ion mass spectrometry (SIMS). In the case of analyzing the sensors, the surfaces of sensors were mechanically polished to a depth of about 0.5 µm before SIMS analysis. The defect distribution in each molecular ion implantation projection range was observed by transmission electron microscopy (TEM) (H-9000UHR-I, Hitachi, Tokyo, Japan). The amounts of molecular ion implantation defects were evaluated by room-temperature photoluminescence (RTPL) analysis (MPL300, WaferMasters, Dublin, CA, USA). The morphology and the distribution of carbon and phosphorus in the CH_2_P-molecular-ion-implanted region at the atomic level were analyzed by laser-assisted atom probe tomography (L-ATP) (LEAP 4000XSi, AMETEK, Berwyn, PA, USA). The L-ATP map and distribution of each element were analyzed using integrated visualization and analysis software (IVAS) from CAMECA (Gennevilliers, France). Finally, the carrier concentration distribution in the depth direction of CH_2_P-molecular-ion-implanted epitaxial silicon wafers were measured by spreading resistance analysis (SRA) (SSM SPR 2000, Semilab, Budapest, Hungary).

## 3. Results

### 3.1. Gettering Capability of CH_2_P-Molecular-Ion-Implanted Epitaxial Silicon Wafer

**Figure 3:**
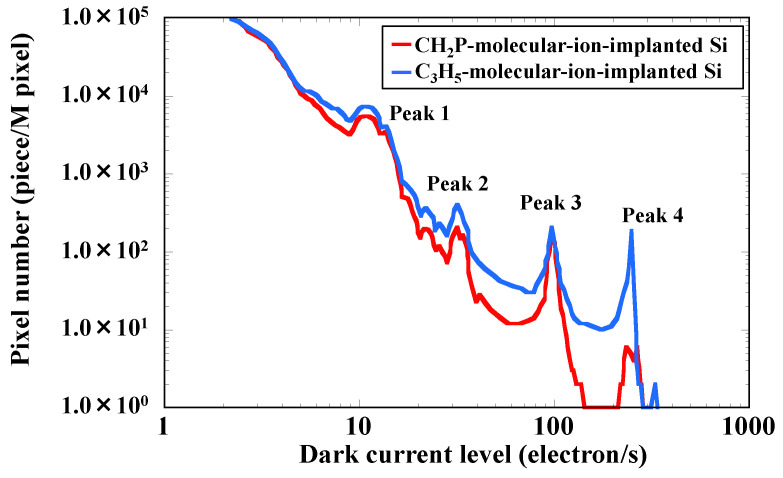
Dark current histogram obtained by DCS at 60 °C for CMOS image sensors fabricated with CH_2_P- and C_3_H_5_-molecular-ion-implanted epitaxial silicon wafers.

Figure 3 shows the DCS spectra of the sensors fabricated with CH_2_P- and C_3_H_5_-molecular-ion-implanted epitaxial silicon wafers measured at 60 °C. Our previous study already reported the dark current amount of CMOS image sensor dependence on, before, and after molecular ion implantation, such as C_3_H_5_ and CH_3_O molecular ions using DCS [26,34]. As a result, the molecular ion implantation technique can drastically decrease the dark current amount during the CMOS image sensor fabrication process. These previous study results indicate that the molecular-ion-implanted epitaxial silicon wafer has a higher metallic impurity gettering capability compared with the conventional epitaxial silicon wafer.

Furthermore, it is well known that there are three components of dark current at the photo–diode junction in CMOS image sensor pixels. Dark current (I_dark_) is forming the generation current (I_generation_), the surface generation current (I_surface_), and the diffusion current (I_diffusion_) (where I_dark_ = dark current + white spot defects = I_generation_ + I_surface_ + I_diffusion_). The first two components are related to the process-induced defects, such as metallic impurity, deep-level defect concentration, SiO_2_/Si interface state defect concentration in the photo–diode space charge region, the transfer gate transistor in CMOS image sensor pixels, and last component is related to the energy band gap of intrinsic semiconductor silicon material using CMOS image sensor fabrication.

In the case of the 60 °C dark current measurement condition in this study, the I_dark_ dominant component is I_generation_. This is because I_diffusion_ depends on an intrinsic semiconductor physical constant such as energy band gap, and I_surface_ does not depend on dark current measurement temperature.

We found that the DCS spectra have four peaks. The three peaks (Peaks 1, 2, and 4) of the DSC spectrum of the sensor with the CH_2_P-molecular-ion-implanted region are lower than those of the sensor with the C_3_H_5_-molecular-ion-implanted region. In particular, Peak 4 is significantly lower. In contrast, Peak 3 is not markedly different between the two spectra. Thus, it is considered that Peak 3 corresponds to dark current from process-induced defects rather than from metallic-impurity-related defects [26].

**Figure 4:**
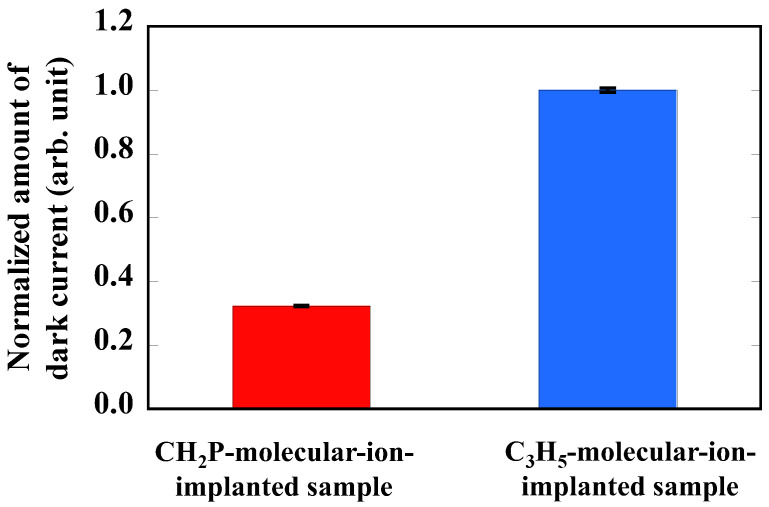
Normalized amount of dark current for CMOS image sensors fabricated with CH_2_P- and C_3_H_5_-molecular-ion-implanted epitaxial silicon wafers.

Figure 4 shows the normalized amount of dark current of CMOS image sensors fabricated by the epitaxial silicon wafers with the CH_2_P- and C_3_H_5_-molecular-ion-implanted region, as determined from the DCS spectra shown in Figure 3. The amount of dark current is defined as the cumulative number of pixels, which is detected as the amount of generated electrons shown in high dark current levels, exceeding 35 electron/s as determined from the DCS spectra. The amount of dark current in the sensors with the CH_2_P-molecular-ion-implanted region is 67% smaller than that in the sensors with the C_3_H_5_-molecular-ion-implnated region. We consider that the difference in the ratio of the amount of dark current depends on the metallic impurity concentration localized in the pixels. Thus, the analysis results focusing on the gettering capability of sensor wafers with the CH_2_P- and C_3_H_5_-molecular-ion-implanted regions are shown below to clarify the reduction in metallic impurities-related defects in the CMOS image sensor active region.

**Figure 5:**
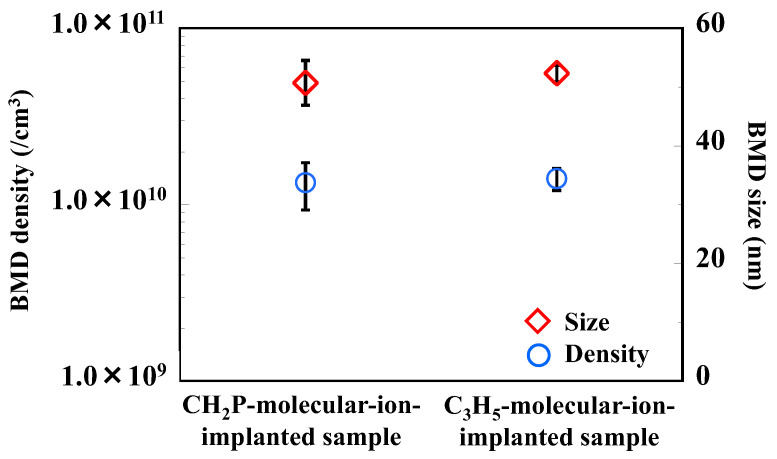
Cross-sectional BMD density and size determined by optical microscopy observant ion of CMOS images sensor fabricated with CH_2_P- and C_3_H_5_-molecular-ion-implanted epitaxial silicon wafers.

Figure 5 shows the size and density of BMDs formed in the silicon substrate of the CMOS image sensors with CH_2_P- and C_3_H_5_-molecular-ion-implanted epitaxial silicon wafers. Both CMOS image sensors were fabricated using carbon-doped silicon wafer substrates with high densities of BMD acting as intrinsic gettering (IG) sites for metallic impurities [35,36]. The CMOS image sensors show no significant differences in BMD size and density after the device fabrication process. IG capability depends on the BMD size and density. Thus, the IG capability is not significantly different between the sensors with CH_2_P and C_3_H_5_ molecular ion implantation. This finding indicates that the reduction in the amount of dark current depends on molecular ion implantation conditions such as the molecular ion species.

**Figure 6:**
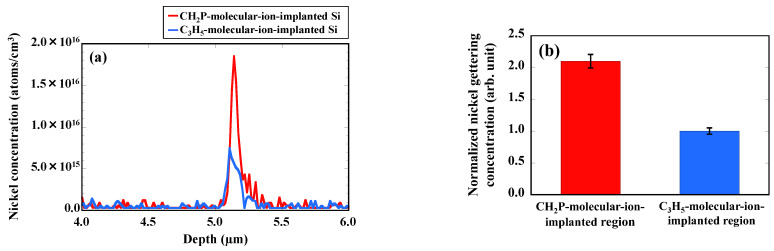
(**a**) SIMS depth profiles of concentration of gettered nickel impurities in CH_2_P- and C_3_H_5_-molecular-ion-implened regions of CMOS image sensors. (**b**) Normalized amount of nickel gettered in CH_2_P- and C_3_H_5_-molecular-ion-implaned regions after CMOS image sensor fabrication process.

Figure 6a shows the depth profiles of the concentration of nickel metallic impurities in CMOS image sensors with the CH_2_P- and C_3_H_5_-molecular-ion-implantated region. The nickel impurities are gettered in each molecular-ion-implanted region formed under the epitaxial silicon layer. The amount of nickel impurities gettered in the CH_2_P-molecular-ion-implanted region is twice as high as that gettered in the C_3_H_5_-molecular-ion-implanted region, as shown Figure 6b. The amount of dark current and the amount of gettered nickel metallic impurities show opposite trend tendencies. Thus, the amount of dark current is reduced depending on the gettering capability of the molecular-ion-implanted region. Therefore, we then focused our investigation on the differences in the morphology of implantation defects, and concentrations of carbon and phosphorus between CH_2_P- and C_3_H_5_-molecular-ion-implanted epitaxial silicon wafers.

### 3.2. Characteristics of CH_2_P-Molecular-Ion-Implanted Region after Epitaxial Growth

**Figure 7:**
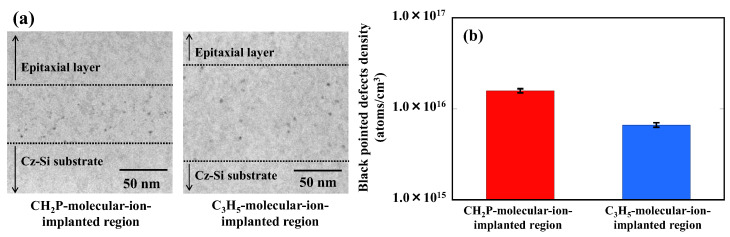
(**a**) Cross-sectional TEM images of CH_2_P- and C_3_H_5_-molecular-ion-implanted regions with epitaxial silicon wafers. (**b**) Density of black pointed defects distributed in CH_2_P- and C_3_H_5_-molecular-ion-implanted regions.

Figure 7a,b show cross-sectional TEM images of the CH_2_P- and C_3_H_5_-molecular ion-implanted regions in epitaxial silicon wafers. Both molecular-ion-implanted regions showed only black pointed defects, and no CH_2_P-molecular-ion-implantation-related specific defects were observed. However, the width of the distribution of black pointed defects in the CH_2_P-molecular-ion-implanted region is 60 nm, which is smaller than that of 100 nm in the C_3_H_5_-molecular-ion-implanted region. From these results, the densities of black pointed defects distributed in the CH_2_P- and C_3_H_5_-molecular-ion-implanted regions are 1.58 × 10^16^ and 6.67 × 10^15^/cm^3^, respectively. Thus, the black pointed defects in the CH_2_P-molecular-ion-implanted region distribute more locally than those in the C_3_H_5_- molecular-ion-implanted region.

**Figure 8:**
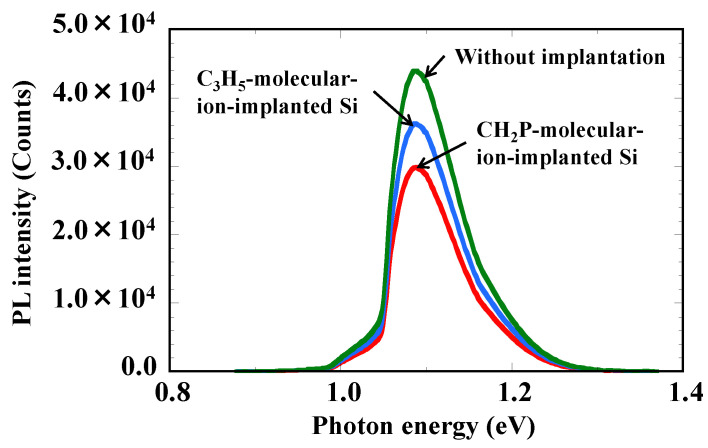
RTPL spectra under 827 nm excitation in epitaxial silicon wafers without and with CH_2_P- and C_3_H_5_-molecular-ion-implanted regions.

Figure 8 shows RTPL spectra under 827 nm excitation in the epitaxial silicon wafers without and with the CH_2_P- and C_3_H_5_-molecular-ion-implanted regions. The penetration depth of 827 nm excitation is around 10 µm, which reflects the PL emission intensity in regions including the molecular-ion-implanted region. The interband transition emission peak intensity of silicon (1.12 eV) in both the CH_2_P- and C_3_H_5_-molecular-ion-implanted epitaxial silicon wafers is lower than that in the silicon wafer without molecular ion implantation. In addition, the intensity in the CH_2_P-molecular-ion-implanted epitaxial silicon wafer is lower than that in the C_3_H_5_-molecular-ion-implanted epitaxial silicon wafer. Thus, this experimental result indicates that the amount of implantation defects in the CH_2_P-molecular-ion-implanted region is higher than that in the C_3_H_5_-molecular-ion-implanted region.

**Figure 9:**
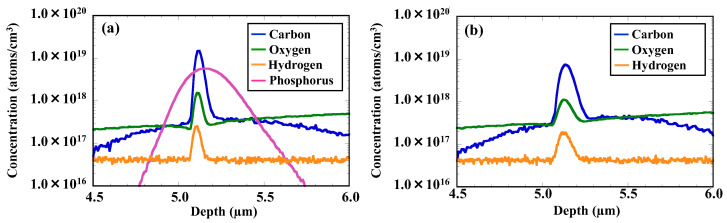
SIMS depth profiles of concentration of (**a**) CH_2_P- and (**b**) C_3_H_5_-molecular-ion-implanted regions after epitaxial growth.

Figure 9a,b show SIMS depth profiles of various element concentrations in the CH_2_P- and C_3_H_5_-molecular-ion-implanted regions after epitaxial growth. The concentrations of phosphorus and carbon are high in the CH_2_P-molecular-ion-implanted region after epitaxial growth. The depth profiles of carbon concentration in the CH_2_P-molecular-ion-implanted region show a higher peak and a sharper distribution than those in the C_3_H_5_-molecular-ion-implanted region. Oxygen impurities that diffuse to the epitaxial layer from the silicon substrate are trapped in both molecular-ion-implanted regions. Moreover, hydrogen is also trapped in both molecular-ion-implanted regions. The trapped hydrogen diffuses during the CMOS device fabrication process and acts as the passivation effect for D_it_ at the SiO_2_/Si interface [21,22,23,24].

**Figure 10:**
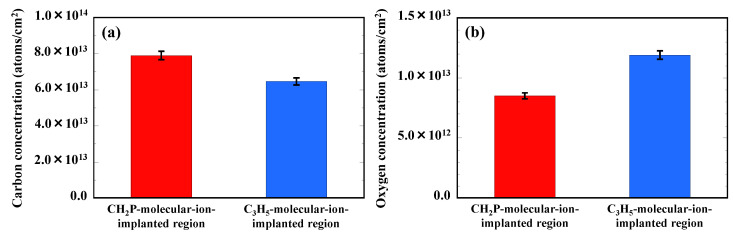
(**a**) Carbon and (**b**) oxygen concentrations localized in CH_2_P- and C_3_H_5_-molecular-ion-implanted regions after epitaxial growth.

Figure 10a,b show the carbon and oxygen concentrations localized in the CH_2_P- and C_3_H_5_-molecular-ion-implanted regions, which are obtained from Figure 9a,b. The carbon concentration of in the CH_2_P-molecular-ion-implanted region is higher than that in the C_3_H_5_-molecular-ion-implanted region after epitaxial growth regardless of the same carbon ion implantation dose. On the other hand, the concentration of oxygen impurities trapped in the CH_2_P-molecular-ion-implanted region is lower than that in the C_3_H_5_-molecular-ion-implanted region. These findings indicate that carbon localized in the CH_2_P-molecular-ion-implanted region has less interaction with oxygen, such as the formation of the C–O complex.

**Figure 11:**
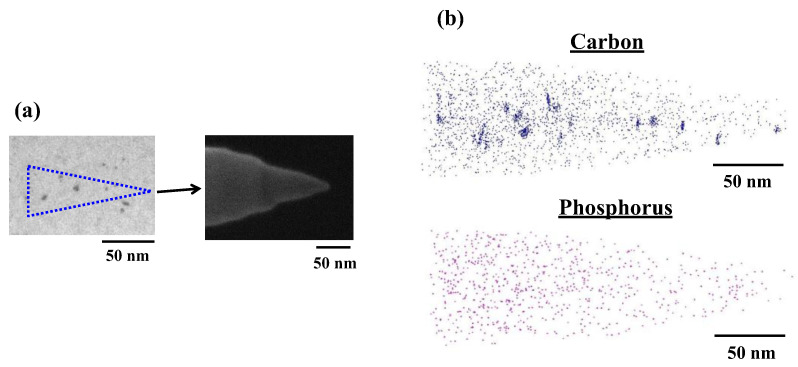
(**a**) TEM (left) and SEM (right) images of acicular sample for L-ATP. (**b**) 3D-APT map of carbon and phosphorus in CH_2_P-molecular-ion-implanted region after epitaxial growth.

Figure 11a shows scanning electron microscopy (SEM) images of an acicular sample for L-APT in the CH_2_P-molecular-ion-implanted region after epitaxial growth. The acicular sample was machined parallel to the molecular-ion-implanted region, focusing on the black-pointed defects using a focused ion beam. Figure 11b shows the 3D distributions map of carbon (blue) and phosphorus (pink) in the CH_2_P-molecular-ion-implnted region in the epitaxial silicon wafer determined by L-APT. Carbon atoms locally agglomerate in the molecular-ion-implanted region, and correspond to the black pointed defects observed in the TEM image. Carbon atoms probably delineate the aggregations of C–I clusters consisting of carbon and silicon self-interstitials. On the other hand, phosphorus atoms are uniformly distributed in the CH_2_P-molecular-ion-implanted region. Thus, from the 3D distribution map, it is considered that agglomerated carbon and phosphorus atoms are not synchronized and do not from complexes.

**Figure 12:**
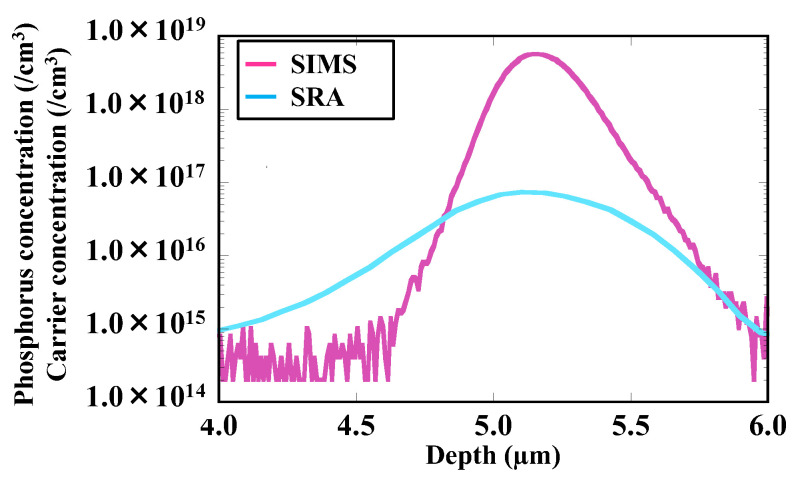
Superposition of depth profiles of phosphorus and carrier concentrations in CH_2_P-molecular-ion-implanted region after epitaxial growth.

Figure 12 shows the superposition of depth profiles of the phosphorus and carrier concentrations in the CH_2_P-molecular-ion-implanted region after epitaxial growth obtained by SIMS and SRA. The peak concentration of phosphorus is 5.70 × 10^18^/cm^3^, whereas the peak carrier concentration is 7.37 × 10^16^/cm^3^. Thus, 98% phosphorus are deactivated. Previous studies showed that the deactivated phosphorus form complexes with vacancies such as P_n_–V [37,38].

## 4. Discussion

### 4.1. Origin of Specific Gettering Sinks in CH_2_P-Molecular-Ion-Implanted Region

We examine why the gettering capability of the CH_2_P-molecular-ion-implanted region is higher than that of the C_3_H_5_-molecular-ion-implanted region at the same carbon dose. From the evaluation results of each molecular-ion-implanted epitaxial silicon wafer, the characteristics of the CH_2_P-molecular-ion-implanted region are summarized as follows in comparison with those of the C_3_H_5_-molecular-ion-implanted region:

(1) The density of black pointed defects distributed in the CH_2_P-molecular-ion-implanted region is higher than that in the C_3_H_5_-molecular-ion-implanted region;
(2) The carbon concentration localized in the CH_2_P-molecular-ion-implanted region is higher than that in the C_3_H_5_-molecular-ion-implanted region; and
(3) Phosphorus is deactivated by forming P_n_–V complexes and does not interact with carbon distributed at the same depth in the CH_2_P-molecular-ion-implanted region.

### 4.2. Formation Model of Gettering Sinks in CH_2_P-Molecular-Ion-Implanted Region

**Figure 13:**
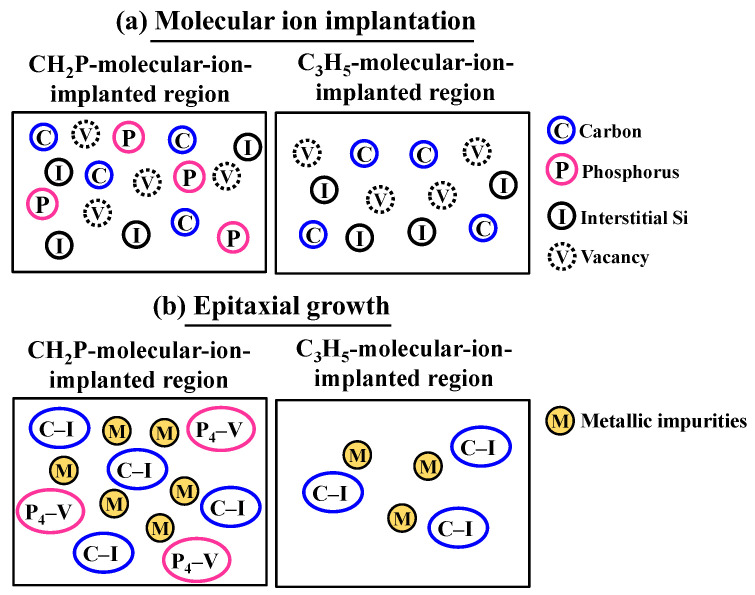
Illustration of formation model of each complex in CH_2_P- and C_3_H_5_-molecular-ion-im- planted regions during (**a**) molecular ion implantation and (**b**) epitaxial growth.

First, we describe the formation model of gettering sinks in the CH_2_P- and C_3_H_5_-molecular-ion-implanted regions, as shown in Figure 13. In the case of CH_2_P molecular ion implantation, carbon and phosphorus are implanted into the silicon surface; and at the same time, Frenkel pairs, such as interstitial silicon and vacancies, are generated. Then, the implanted carbon and phosphorus form a complex by reacting with the Frenkel pairs during epitaxial growth as follows:

C + I_Si_ → C–I

P_n_ + V → P_n_–V

**Figure 14:**
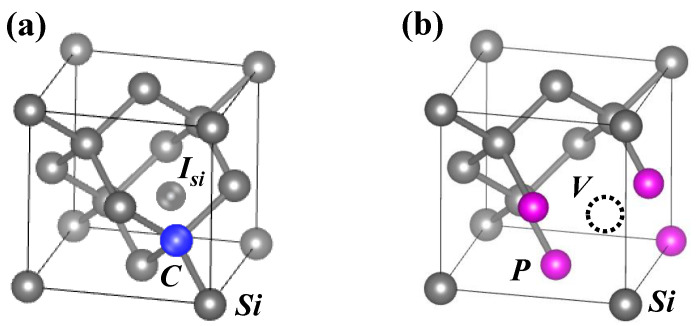
Schematics of (**a**) C–I and (**b**) P_4_–V complex formed in CH_2_P-molecular-ion-implanted region after epitaxial growth. The carbon and phosphorus are blue and pink, respectively.

I_Si_ + V → Si_s_

where C is carbon, I_Si_ is interstitial silicon, P is phosphorus (*n* = 1–4), V is vacancy, and Si_s_ is substitutional silicon. Carbon interacts with interstitial silicon to form a C–I complex, and phosphorus interacts with vacancies to form a P_n_–V complex such as the P_4_–V complex, as shown in Figure 14a,b. The probability of annihilation with interstitial silicon and vacancies is low.

Pawlak and Duffy investigated the co-implantation of carbon and phosphorus monomer ions to suppress the enhanced phosphorus diffusion due to interact with interstitial silicon [39]. They showed that carbon and interstitial silicon generated during co-implantation predominantly form a C–I complex; thereby, the enhanced phosphine diffusion was suppressed. It is considered that the same reaction occurs in the CH_2_P-molecular-ion-implanted region after epitaxial growth. On the other hand, the concentration of phosphorus is not high in the C_3_H_5_-molecular-ion- implanted region. Only the C–I complex is formed, and there is a high possibility that interstitial silicon and vacancies will be annihilated. Carbon that could not interact with interstitial silicon to form a C–I complex diffuses isotropically. Since the density of the C–I complex is low in the C_3_H_5_-molecular-ion-implanted region, the carbon concentration and black pointed defect density are low, as shown by SIMS and TEM. Therefore, the CH_2_P-molecular-ion-implanted region forms two types of complex, namely C–I and P_n_–V, particularly for carbon and phosphorus.

### 4.3. Gettering Capability of These Complexes Distributed in CH_2_P-Molecular-Ion-Implanted Region for Metallic Impurities

Next, we consider the gettering capability of the CH_2_P-molecular-ion-implanted region including C–I and P_n_–V complexes for metallic impurities. Kurita and coworkers described that the gettering sinks in the C_3_H_5_-molecular-ion-implanted region originated from the black-pointed defects, which consist of carbon complexes such as agglomerated carbon–silicon self-interstitial clusters (C–I complex) [19,20,25,26]. The C–I complex has been shown by density functional theory (DFT) calculation to have high binding energies to metallic impurities and acts as a strong gettering sink [40,41,42]. Moreover, Masada and coworkers also concluded that the gettering capability of agglomerated C–I complexes for metallic impurities depends on the oxygen concentration in agglomerated C–I complexes, and that agglomerated C–I complexes with low oxygen concentrations have a high gettering capability, as shown by electron interaction with metallic impurities and nanostructure analysis using L-ATP [43,44,45]. SIMS analysis results show a similar tendency of the carbon and oxygen concentrations localized in the CH_2_P-molecular-ion-implanted region after epitaxial growth.

As for the P_n_–V complex, Chan et al. developed gettering models of transition metals in the high-phosphorus-concentration region using DFT [46]. Their results showed that the P_n_–V complex strongly binds to transition metals. In particular, the critical complex responsible for both phosphorus deactivation and metal gettering was identified to be the P_4_–V complex that most strongly binds transition metals, as shown Figure 14b.

Therefore, the CH_2_P-molecular-ion-implanted epitaxial silicon wafer has a high gettering capability for metallic impurities because this implanted region is formed with high densities of agglomerated C–I complexes and P_4_–V complexes.

## 5. Conclusions

We investigated the amount of dark current in CMOS image sensors fabricated with CH_2_P-molecular-ion-implanted epitaxial silicon wafers. The amount of dark current in the CMOS image sensor with the CH_2_P-molecular-ion-implanted region was 67% lower than that of the CMOS image sensor with the C_3_H_5_-molecular-ion-implanted region with the same carbon dose. The CH_2_P-molecular-ion-implanted epitaxial silicon wafers show the same three characteristics as the C_3_H_5_-molecular-ion-implanted epitaxial silicon wafers that can resolve the three important technological issues of 3D-CISs. Among them, we specifically found the improvement of the gettering capability for metallic impurities. Focusing on the gettering capability of the CH_2_P-molecular-ion-implanted region, the amount of nickel impurities gettered in the CH_2_P-molecular-ion-implanted region was twice that gettered in the C_3_H_5_-molecular-ion-implanted region after the CMOS image sensor fabrication process. Regarding the characteristics of the CH_2_P-molecular-ion-implanted region, the carbon peak concentration and black pointed defect density are high, and phosphorus is mainly distributed in a deactivated state.

Therefore, the CH_2_P-molecular-ion-implanted region has two types of gettering sink, namely, the high-density C–I complex and P_4_–V complex aggregates, which have a high binding energy for metallic impurities. We believe that CH_2_P-molecular-ion-implanted epitaxial silicon wafers can contribute to the improvement of the performance of CMOS image sensors.

## Data Availability

This data presented in this study are available on request from the corresponding author. The data are not publicly available because of confidentiality concerns.

## References

[1] Mizuta K., Tsugawa H., Nakamura R., Kagawa Y., Takahashi T., Sakakibara M., Tatani K. (2019). The Evolutionary Process for 3D Stacked CMOS Image Sensor and the Advanced Technologies. Vac. Surf. Sci..

[2] Ohta J. (2015). Smart CMOS Image Sensors and Application.

[3] Bea J., Lee K., Fukushima T., Tanaka T., Koyanagi M. (2011). Evaluation of Cu Diffusion from Cu Through-Silicon via (TSV) in Three-Dimensional LSI by Transient Capacitance Measurement. IEEE Electron Device Lett..

[4] Lee K., Bea J., Ohara Y., Murugesn M., Fukushima T., Tanaka T., Koyanagi M. (2014). Impact of Cu Contamination on Device Reliabilities in 3-D IC Integration. IEEE Trans. Device Mater. Reliab..

[5] Kagawa Y., Fujii N., Aoyagi K., Kobayashi Y., Nishi S., Takeshita S., Taura J., Takahashi H., Tatani K., Kawamura M. An Advanced CuCu Hybrid Bonding For Novel Stacked CMOS Image Sensor. Proceedings of the IEEE Electron Device Technology and Manufacturing Conference.

[6] Graff K. (2000). Metal Impurities in Silicon-Devices Fabrication.

[7] Russo F., Nardone G., Polignano M.L., D’Ercole A., Pennella F., Felico M.D., Monta A.D., Matarazzo A., Moccia G., Polsinella G. (2017). Dark Current Spectroscopy of Transition Metals in CMOS Image Sensors. ECS J. Solid State Sci. Technol..

[8] Shoyama T. Influence of various impurities on performance of CMOS image sensor. Proceedings of the 2018 8th Forum on the Science and Technology of Silicon Materials.

[9] Tournier A., Leverd F., Favennec L., Perrot C., Pinzelli L., Gatefait M., Cherault N., Jeanjean D., Carrere J.–P., Hirigoyen F. Pixel-to-Pixel isolation by Deep Trench technology: Application to CMOS Image Sensor. Proceedings of the 2011 International Image Sensor Workshop.

[10] Caplan P.J., Poindexter E.H. (1979). ECR centers, interface states, and oxide fixed charge in thermally oxidized silicon wafers. J. Appl. Phys..

[11] Poindexter E.H., Gerardi G.J., Rueckel M.-E., Caplan P.J. (1984). Electronic traps and P_b_ centers at the Si/SiO_2_ interface: Band-gap energy distribution. J. Appl. Phys..

[12] Regolini J.L., Benoit D., Morin P. (2007). Passivation issues in active pixel CMOS image sensors. Microelectron. Reliab..

[13] Benoit D., Regolini J.L., Morin P. (2007). Hydrogen desorption and diffusion in PECVD silicon nitride. Application to passivation of CMOS active pixel sensors. Microelectron. Reliab..

[14] Chen P.J., Wallace R.M. (1999). Deuterium transport through device structures. J. Appl. Phys..

[15] Yamade I., Matsuo J. (1997). Solid surface process by gas cluster ion beam. Oyo Buturi.

[16] Tanjyo M., Hamamoto N., Nagayama T., Umisedo S., Koga Y., Maehara N., Une H., Matsumoto T., Nagai N., Borland J.O. (2009). Cluster Ion Implantation system: Claris for Beyond 45 nm Device Fabrication (II). ECS Trans..

[17] Kadono T., Kurita K. (2015). Method of Producing Semiconductor Epitaxial Wafer, Semiconductor Epitaxial Wafer, and Method of Producing Solid-State Image Sensing Device. Japan Patent.

[18] Kurita K. (2015). Progress of silicon wafer gettering technology. Oyo Buturi.

[19] Kurita K., Kadono T., Okuyama R., Hirose R., Onaka-Masada A., Koga Y., Okuda H. (2016). Proximity gettering of C3H5 carbon cluster ion-implanted silicon wafers for CMOS image sensors: Gettering effects of transition metal, oxygen, and hydrogen impurities. Jpn. J. Appl. Phys..

[20] Kurita K., Kadono T., Okuyama R., Shigematsu S., Hirose R., Onaka-Masada A., Koga Y., Okuda H. (2017). Proximity Gettering Technology for Advanced CMOS image sensors using carbon cluster ion-implantation technique: A review. Phys. Status Solid A.

[21] Okuyama R., Kadono T., Masada A., Hirose R., Koga Y., Okuda H., Kurita K. (2017). Trapping and diffusion kinetic of hydrogen in carbon-cluster ion-implantation projected range in Czochralski silicon wafers. Jpn. J. Appl. Phys..

[22] Okuyama R., Shigematsu S., Hirose R., Masada A., Kadono T., Koga Y., Okuda H., Kurita K. (2017). Trapping and diffusion behavior of hydrogen simulated with TCAD in projection range of carbon-cluster implanted silicon epitaxial wafers for CMOS image sensors. Phys. Status Solidi C.

[23] Okuyama R., Kadono T., Onaka-Masada A., Suzuki A., Kobayashi K., Shigematsu S., Hirose R., Koga Y., Kurita K. (2020). Hydrogen passivation for reduction of SiO_2_/Si interface state density using hydrocarbon-molecular-ion-implanted silicon wafers. Jpn. J. Appl. Phys..

[24] Okuyama R., Kadono T., Onaka-Masada A., Suzuki A., Kobayashi K., Shigematsu S., Hirose R., Koga Y., Kurita K. (2022). Hydrogen diffusion behavior in CH2P-molecuar-ion-implanted silicon wafers for CMOS image sensors. Mater. Sci. Semicond. Process..

[25] Kurita K., Kadono T., Okuyama R., Shigematsu S., Hirose R., Onaka-Masada A., Koga Y., Okuda H. (2019). A Review of Proximity Gettering Technology for CMOS Image Sensors Using Hydrocarbon Molecular Ion Implantation. Sens. Mater..

[26] Kurita K., Kadono T., Shigematsu S., Hirose R., Okuyama R., Onaka-Masada A., Okuda H., Koga Y. (2019). Proximity Gettering Design of Hydrocarbon-Molecular-Ion-Implanted Silicon Wafers Using Dark Current Spectroscopy for CMOS Image Sensors. Sensors.

[27] McGrath R.D., Doty J., Lupino G., Ricker G., Vallerga V. (1987). Counting of Deep-Level Traps Using a Charge-Coupled Devices. IEEE Trans. Electron Devices.

[28] Mccolgin W.C., Lavine J.P., Stancampiano C.V. (1995). Probing metal Defects in CCD Image Sensors. MRS Proc..

[29] Mccolgin W.C., Lavine J.P., Stancampiano C.V. (1996). Dark Current Spectroscopy of Metals in Silicon. MRS Proc..

[30] Shimura F. (1989). Semiconductor Silicon Crystal Technology.

[31] Rozgoni G.A., Petroff P.M., Read M.H. (1975). Elimination of Oxidiation-Induced Stacking Faults by Preoxidation Gettering of Silicon wafers. J. Electrochem. Soc..

[32] Tseng W.F., Koji T., Mayer J.W., Seidel T.E. (1987). Simultaneous gettering of Au in Silicon by phosphorus and dislocations. Appl. Phys. Lett..

[33] Lecrosnier D., Paugam J., Richou F., Pelous G., Beniere F. (1980). Influence of phosphorus-induced point defects on a gold-gettering mechanism in silicon. J. Appl. Phys..

[34] Hirose R., Kadono T., Okuyama R., Onaka-Masada A., Shigematsu S., Kobayashi K., Koga Y., Kurita K. (2019). Proximity gettering technique using CH_3_O multielement molecular ion implantation for the reduction of the white spot defect density in CMOS image sensor. Jpn. J. Appl. Phys..

[35] Sueoka K., Sadamitsu S., Koike Y., Kihara T., Katahama H. (2000). Internal Gettering for Ni Contamination in Czochralski Silicon Wafers. J. Electrochem. Soc..

[36] Sueoka K. (2005). Modeling of Internal Gettering of Nickel and Copper by Oxide Precipitates in Czochralski-Si Wafers. J. Electrochem. Soc..

[37] Takamura Y., Jain S.H., Griffin P.B., Plummer J.D. (2002). Thermal Stability of dopants in laser annealed silicon. J. Appl. Phys..

[38] Takamura Y., Griffin P.B., Plummer J.D. (2002). Physical processes associated with the deactivation of dopants in laser annealed silicon. J. Appl. Phys..

[39] Pawlak B.J., Duffy R. (2006). Suppression of phosphorus diffusion by carbon co-implantation. Appl. Phys. Lett..

[40] Jin Y., Dunham S.T. (2014). Modeling of Carbon Clustering and Associated Metal Gettering. ECS Trans..

[41] Shirasawa S., Sueoka K., Yamaguchi T., Maekawa K. (2015). Useful Database of Effective Gettering Sites for Metal Impurities in Si Wafers with First Principle Calculation. J. Electrochem. Soc..

[42] Shirasawa S., Sueoka K., Yamaguchi T., Maekawa K. (2016). Density functional theory calculations for estimation of gettering sites of C, H, intrinsic point defects and related complexes in Si wafers. Mater. Sci. Semicond. Process..

[43] Onaka-Masada A., Nakai T., Okuyama R., Okuda H., Kadono T., Hirose R., Koga Y., Kurita K., Sueoka K. (2018). Effect of Low-oxygen-concentration layer on iron gettering capability of carbon-cluster ion-implanted Si wafer for CMOS image sensors. Jpn. J. Appl. Phys..

[44] Onaka-Masada A., Kadono T., Okuyama R., Hirose R., Kobayashi K., Suzuki A., Koga Y., Kurita K. (2020). Reduction of Dark Current in CMOS Image Sensor Pixels Using Hydrocarbon-Molecular-Ion-Implanted Double Epitaxial Si Wafers. Sensors.

[45] Shigematsu S., Okuyama R., Hirose R., Kadono T., Onaka-Masada A., Suzuki A., Kobayashi K., Okuda H., Koga Y., Kurita K. (2020). Influence of oxygen on copper gettering in hydrocarbon molecular ion implanted region using atom probe tomography. Nucl. Instrum. Methods Phys. Res. B.

[46] Chen R., Trzynadlowski B., Dunham S.T. (2014). Phosphorus vacancy cluster model for phosphorus diffusion gettering of metals in Si. J. Appl. Phys..

